# A Novel Function of the Lysophosphatidic Acid Receptor 3 (LPAR3) Gene in Zebrafish on Modulating Anxiety, Circadian Rhythm Locomotor Activity, and Short-Term Memory

**DOI:** 10.3390/ijms21082837

**Published:** 2020-04-18

**Authors:** Yu-Nung Lin, Gilbert Audira, Nemi Malhotra, Nguyen Thi Ngoc Anh, Petrus Siregar, Jen-Her Lu, Hsinyu Lee, Chung-Der Hsiao

**Affiliations:** 1 Department of Life Science, National Taiwan University, Taipei 11221, Taiwan; d01b41008@ntu.edu.tw; 2 Department of Chemistry, Chung Yuan Christian University, Chung-Li 32023, Taiwan; gilbertaudira@yahoo.com; 3 Department of Bioscience Technology, Chung Yuan Christian University, Chung-Li 32023, Taiwan; nguyen021194@gmail.com (N.T.N.A.); siregar.petrus27@gmail.com (P.S.); 4 Department of Biomedical Engineering, Chung Yuan Christian University, Chung-Li 32023, Taiwan; nemi.malhotra@gmail.com; 5 Department of Pediatrics, Taipei Veterans General Hospital, Taipei 11221, Taiwan; jenherlu@gmail.com; 6 School of Medicine, National Yang-Ming University, Taipei 11221, Taiwan; 7 School of Medicine, National Defense Medical Center, Taipei 11490, Taiwan; 8 Center for nanotechnology, Chung Yuan Christian University, Chung-Li 32023, Taiwan

**Keywords:** zebrafish, anxiety, memory, circadian rhythm locomotor activity, behavior, lysophosphatidic acid receptor

## Abstract

Lysophosphatidic acid (LPA) is a small lysophospholipid molecule that activates multiple cellular functions through pathways with G-protein-coupled receptors. So far, six LPA receptors (LPAR1 to LPAR6) have been discovered and each one of them can connect to the downstream cell message-transmitting network. A previous study demonstrated that LPA receptors found in blood-producing stem cells can enhance erythropoietic processes through the activation of LPAR3. In the current study, newly discovered functions of LPAR3 were identified through extensive behavioral tests in *lpar3* knockout (KO) zebrafish. It was found that the adult *lpar3* KO zebrafish display an abnormal movement orientation and altered exploratory behavior compared to that of the control group in the three-dimensional locomotor and novel tank tests, respectively. Furthermore, consistent with those results, in the circadian rhythm locomotor activity test, the *lpar3* KO zebrafish showed a lower level of angular velocity and average speed during the light cycles, indicating an hyperactivity-like behavior. In addition, the mutant fish also exhibited considerably higher locomotor activity during the dark cycle. Supporting those findings, this phenomenon was also displayed in the *lpar3* KO zebrafish larvae. Furthermore, several important behavior alterations were also observed in the adult *lpar3* KO fish, including a lower degree of aggression, less interest in conspecific social interaction, and looser shoal formation. However, there was no significant difference regarding the predator avoidance behavior between the mutant and the control fish. In addition, *lpar3* KO zebrafish displayed memory deficiency in the passive avoidance test. These in vivo results support for the first time that the *lpar3* gene plays a novel role in modulating behaviors of anxiety, aggression, social interaction, circadian rhythm locomotor activity, and memory retention in zebrafish.

## 1. Introduction

Lysophosphatidic acid, a phospholipid, is a mediator for many biological and physiological processes such as cell differentiation, cell proliferation, cell-to-cell interactions, smooth muscle contraction, and platelet aggregation [1,2]. LPA is also involved in processes such as cytoskeletal rearrangement [3] and tumorigenesis [4,5]. LPA has been detected in biological fluids in micromolar quantities such as in serum, plasma [6], and follicular fluid [7,8]. Various cells in the body such as endometrial cells [9,10], ovarian cells [11,12], mast cells [13], erythrocytes [14], and neurons [15] have the capability to produce LPA. LPA has been known to be involved in pathological consequences in reproductive tissue associated with tumors [5,16] as well as in severe diseases. Two pathways of LPA production have been established, yet LPA metabolism within most types of cells is still indistinct [5]. Several pathways of LPA synthesis reflect multiple levels of regulation and deregulation in accordance with different physiological and pathological statuses such as cancer [4] and pregnancy [17]. LPA-related genes are found to be highly conserved in vertebrates [18]. 

The LPA receptor (LPAR) is known as a heteromeric G protein-coupled receptor, specifically involved with G(i)/G(o)/G(q). So far, six G protein-coupled receptors have been identified for LPAR [19]. The expression pattern and signaling properties of LPARs are complex and result in multiple influences on physiological, pathological, and developmental processes, which in turn act through G protein-coupled receptors (GPCRs). LPAR mediates several cellular responses such as calcium influx, cell proliferation, and cell survival. LPAR3 was discovered and cloned using homology searches [20], located on human chromosome locus 1p 22.3 and on mouse chromosome locus 3, 71.03 cM, encoding a 353-amino acid sequence GPCR with a molecular mass ~40 kDa [21]. LPAR3 couples with G_αi/o_ and G_αq/11_ to facilitate LPA-induced processes such as calcium mobilization, adenylyl cyclase activation, MAPK activation, and PLC activation. In addition, LPAR also plays critical constructive and destructive roles in disease progression. LPA signaling has been known to participate in development from synchronized regulation of LPA production, degradation, receptor expression, and activity to maintain normal physiological functions, hence, it has received a lot of research attention.

LPAR3 has been proven to be involved in various cellular and physiological processes in the body. It was demonstrated that LPAR3 determines vertebrate left–right patterning during embryogenesis as an important step for proper organ formation and function. In that particular study, downregulation of *lpar3* and inhibition of *lpar3* with Ki16425 interrupted asymmetric gene expression and organ symmetry in zebrafish [22]. Genes related to LPA functions such as LPA receptors (9 genes), LPA-producing enzymes (6 genes), and LPA-degrading enzymes are highly conserved in zebrafish. The amino acid sequence of these LPA-related genes in zebrafish bear 50–70% similarity to their mammalian homologs. Knockdown of the LPA-produced enzyme, autotaxin (ATX), resulted in malformation of embryonic blood-vessel formation in zebrafish embryos, similar to the observation in knockout mice. Therefore, LPA-related genes can be up and downregulated by injecting morpholino antisense oligonucleotide specific to LPA-related genes in zebrafish embryos, for testing various procedures and drugs [18]. In another study, however, activation of *lpar3* suppressed thrombopoiesis in zebrafish, blocked *lpar3* translation by a morpholino increase in the number of CD41-GFP cells in transgenic zebrafish, and increased ZCD41 mRNA expression levels of *lpar3* knockout zebrafish. These results signify the negative impact of *lpar3* during megakaryopoiesis and might help in providing potential treatment of related diseases [23]. In a similar line of study, *lpar3* was established as being critical for megakaryopoiesis and erythropoiesis in zebrafish; here, histological analysis depicted shrinking hematopoietic tissue in kidney marrow of *lpar3*-deficient fish, with no observed morphological differences in other organs. The observation of tissue profiles of *lpar3* KO animals was established as a suitable method for future diagnosis [24].

During the development of neuronal network formation, the activation of LPAR3 has been revealed to induce axonal branching in hippocampal cell cultures mediated through Gq and Rho family GTPase 2 (Rnd2) [25,26]. LPAR has shown diverse cellular responses in cultured astrocytes acting from LPAR1-3 [27,28]. The effects of LPAR on cultured astrocytes include the formation of reactive oxygen species (ROS), calcium immobilization, and decreased uptake of glucose and glutamate, all of which contribute to neurodegeneration [29,30]. LPAR also induces the expression of immediate early genes, cytokine genes, interleukins IL-1b, IL3, IL6, and nerve growth factor [31]. Neurons and astrocytes, functioning simultaneously, are crucial for the normal functioning of the central nervous system. However, the role of LPAR gene on animal behavior, which is mainly controlled by the central nervous system, remains unclear so far. To date, only a few studies have addressed a possible role of LPAR in behavior, and most of these studies only focused on LPAR1 and LPAR5 genes [32,33,34,35,36]. Despite these findings, no studies have tested the involvement of *lpar3* in zebrafish behaviors. Therefore, this study aimed to validate the potential function of the *lpar3* gene in a common animal model of behavior. In the current study, we used zebrafish as a model for pharmacological studies that may provide some relevant insights for the improvement of medications and may also lead to specific directions of research considering the robust, accurate, and fast results from zebrafish. After the behavioral abnormalities in *lpar3* KO fish were observed, several important biomarkers in the fish brain and body were measured to understand the role of this gene in zebrafish behavioral regulation. Finally, *lpar3* KO fish displayed impairments in several behaviors, including hyperactivity-like behavior in the larvae stage and altered exploratory behavior, social interaction, and circadian rhythm locomotor activity in the adult stage. These findings strongly suggest that the LPAR3 receptor is involved in larval and adult zebrafish behavior.

## 2. Results

### 2.1. lpar3 KO Zebrafish Larvae Demonstrated Locomotor Hyperactivity

**Figure 1:**
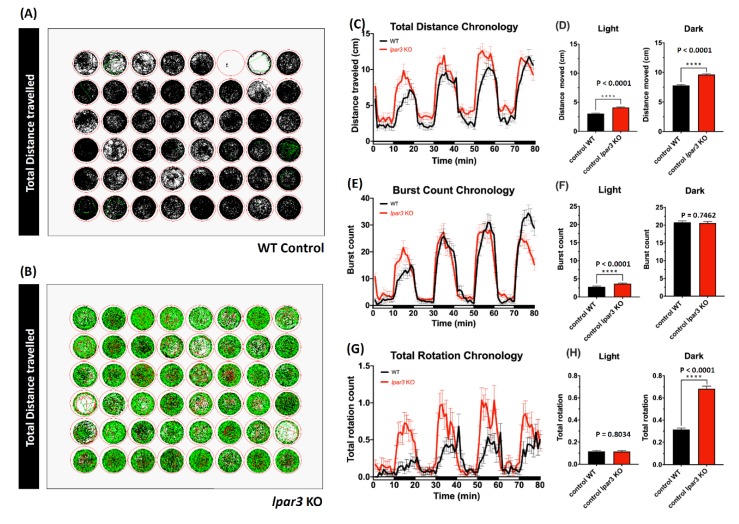
Comparison of behavior endpoints between wild-type and *lpar3* knockout (KO) zebrafish larvae in the light/dark cycle behavior assay. Locomotor tracking for an 80 min recording is presented as a trajectory path for wild-type control (**A**) and *lpar3* KO (**B**). The movements are represented in different colors for different speeds (the black line refers to speeds under 5 mm/s, the green line refers to speeds from 5 to 20 mm/s, and the red line refers to speeds over 20 mm/s). Different endpoints are present in chronological changes of the total distance travelled (**C**), total burst count (**E**), and total rotation (**G**). Total locomotor activities in light and dark cycles were quantified and are presented as a total distance travelled (**D**), total burst count (**F**), and total rotation (**H**). Data are expressed as means ± standard error of the mean (SEM) and significant difference was test by the nonparametric Mann–Whitney test (*n* = 48, **** *p* < 0.0001).

**Figure A1:**
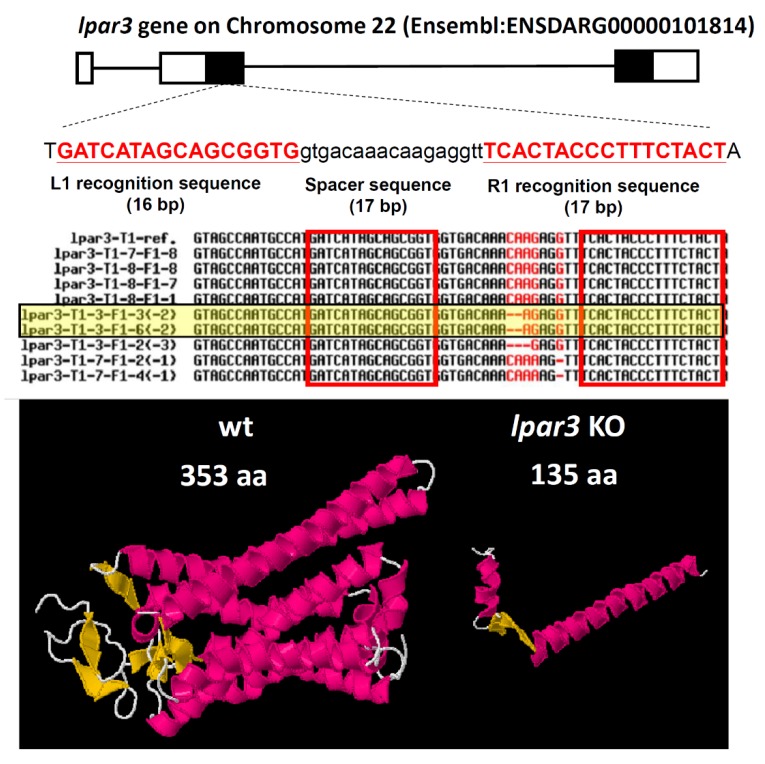
Generation of lysophosphatidic acid receptor 3 (*lpar3*) gene knockout zebrafish by using the transcription activator-like effector nuclease (TALEN) genome editing tool. Scheme shows the TALEN left and right arm sequences used to target the *lpar3* gene on exon 2 in zebrafish (top panel). The left and right arm (highlighted by red color) and spacer sequences used for the TALEN design are also listed. Comparison of the Sanger sequencing results of the F1 generation that carried potential mutations for the *lpar3* gene. The stable *lpar3* KO fish used in this study carrying a 2 nucleotide-deletion (delCA) results in the predicted, truncated LPA3 protein. The predicted three-dimensional structure of the LPAR3 protein for wild-type (wt, containing 353 amino acids) and *lpar3* KO fish (with 135 amino acids). The beta sheet, alpha helix, and random coil protein domain structures are highlighted with yellow, pink, and white colors, respectively.

To study the function of *lpar3*, we generated a zebrafish mutant line carrying *lpar3* gene deficiency by using TALEN-mediated genome editing. This mutant line carries a 2 bp deletion (∆CA) and its corresponding protein was truncated and reduced from 353 to 135 amino acid residues (Figure A1). This *lpar3* KO fish displays an aging phenotype and was reported in our previous publication [37]. In this study, we aimed to explore the potential new function of the *lpar3* gene on behaviors. Initially, we conducted high throughput locomotion monitoring in both *lpar3* KO and wild-type (WT) fish aged at around 5 days-post fertilization (dpf). After moving larvae individually to novel cylindrical wells in 48-well plates, swimming activity was observed with three major endpoints in terms of total distance traveled, burst count, and rotation movement. This assay aimed to show early-stage larvae mobility both temporally and spatially during light and dark phases. Larval swimming behavior was monitored in response to light–dark transitions by using a 10 min interlace of light and dark, and behavior patterns in four repeated cycles were inspected later. Within a total of 80 min of recording, each well contained one larva individually to observe its locomotor response. The locomotor activity was measured with a set of parameters to distinguish different traveling speeds, which were slow movements (expressed in the black-colored line, representing speeds under 5 mm/s), medium speeds (expressed in the green-colored line, representing speeds ranging from 5 to 20 mm/s), and fast movements (expressed in the red-colored line, representing speeds over 20 mm/s.) From the movement trajectory results of wild-type control (Figure 1A) and *lpar3* KO (Figure 1B), it is clear that most of the control larvae swam under 5 mm/s while *lpar3* KO larvae moved significantly faster throughout out both the light and dark cycles. This phenomenon was also shown in other endpoints measured during the test. Based on the total distance chronology, *lpar3* KO larvae showed significantly higher traveling distance both in light and dark phases compared to the wild type (Figure 1C,D), which showed consistency with the higher speed results from total duration. Besides the distance movement, we added abrupt movements, i.e., burst movement counts, as a second endpoint. From these result, *lpar3* KO larvae showed a more robust reaction as the illumination turned from bright and dark, displaying a higher burst count during the light phase compared to the wild type (Figure 1E,F). As for rotation counting, there was significantly higher turning or direction altering during the dark phase observed in *lpar3* KO larvae, indicating hyperactivity-like behavior compared to the wild type (Figure 1G,H).

### 2.2. lpar3 KO Zebrafish Displayed Abnormal Exploratory Behavior in Novel Tank Assay

**Figure 2:**
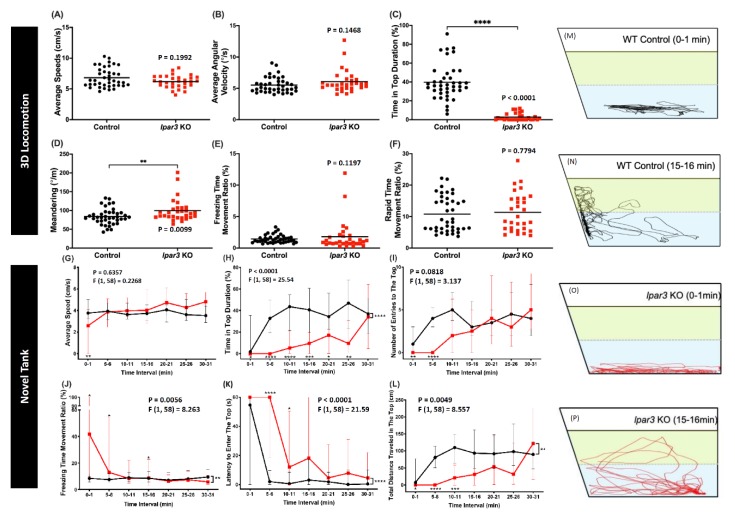
Comparison of behavior endpoints between wild-type (black) and *lpar3* KO (red) fish in 3D locomotion (**A**–**F**) and novel tank (**G**–**P**) tests. In the 3D locomotion test, average speed (**A**), average angular velocity (**B**), time-in-top duration (**C**), meandering (**D**), freezing time movement ratio (**E**), and rapid movement ratio (**F**) were analyzed. The data are expressed as the median and were analyzed by the nonparametric Mann–Whitney test (*n* = 40 for control; *n* = 30 for *lpar3* KO fish). Average speed (**G**), time-in-top duration (**H**), the number of entries to the top (**I**), freezing time movement ratio (**J**), latency to enter the top (**K**), and total distance traveled in the top (**L**) were analyzed during a novel tank test. Locomotor trajectories are expressed in the wild type before (**M**) and after acclimation (**N**) and in *lpar3* KO fish before (**O**) and after acclimation (**P**). The data are expressed as the median with interquartile range and were analyzed by two-way ANOVA with Geisser–Greenhouse correction. To observe the differences between time interval, Sidak’s multiple comparison test was used (*n* = 30 for both groups; * *p* < 0.05, ** *p* < 0.01, *** *p* < 0.001, **** *p* < 0.0001).

After the larvae locomotion tests, adult fish behavior tests were conducted to clarify the relationship between the *lpar3* gene and locomotion. These behavior tests were started with a three-dimensional (3D) locomotor activity test that tracked the detailed trajectories. This assay was reported as a highly sensitive method to evaluate the potential chemical toxicity in fish behavior [38,39]. After a 15-min acclimation in the experiment tank, the *lpar3* KO fish showed similar behavior with the control fish in terms of their locomotor activity as indicated by similar average speed, freezing time movement ratio, and rapid movement ratio (Figure 2A,E,F). However, in terms of their movement orientation, a slight change was detected in the *lpar3* mutant fish. A zigzag-like movement was observed during the test as shown by a high degree of meandering of the mutant fish (Figure 2D), even though there was no significant difference observed regarding their average angular velocity (Figure 2B). In addition, it was found that the mutant fish had altered exploratory behavior, which was indicated by a significantly lower time in the upper level of the water (Figure 2C).

Similar to the rodent open field test, the novel tank test is a method to evaluate zebrafish anxiety levels by testing their innate adaptive ability in a new environment [40]. This ability is measured by the locomotor activity and exploratory behavior of zebrafish. After the locomotor activity was quantified, a slight decrement was found in the mutant fish’s locomotor activity during a 30-min exposure in a novel tank. This was shown by the higher freezing time movement ratio at several time points displayed by the mutant fish compared to the control group (Figure 2J). However, their average speed was not significantly different to that of the control group (Figure 2G). Similar to the 3D locomotion test result, altered exploratory behavior was also displayed by the mutant fish during the test. This alteration was indicated by the low level of time in the top of the tank, low level of total distance traveled in the top of the tank, and high level of latency entering the top of the tank during the test (Figure 2H,L,K). Although there were slight reductions in the number of entries of the mutant fish into the top of the tank at several early time points, there was no significant difference found when compared to that of the control group (Figure 2I). To visualize the differences between the two groups, the trajectories from one-minute of tracking of one fish in each group at different time points are presented. As both groups remained in the bottom region (Figure 2M,O), *lpar3* KO larvae showed a lower duration of staying in the upper tank region after acclimation, which was different from the control group (Figure 2N,P).

### 2.3. lpar3 KO Zebrafish Displayed Less Aggression but Normal Predator Avoidance Behaviors

**Figure 3:**
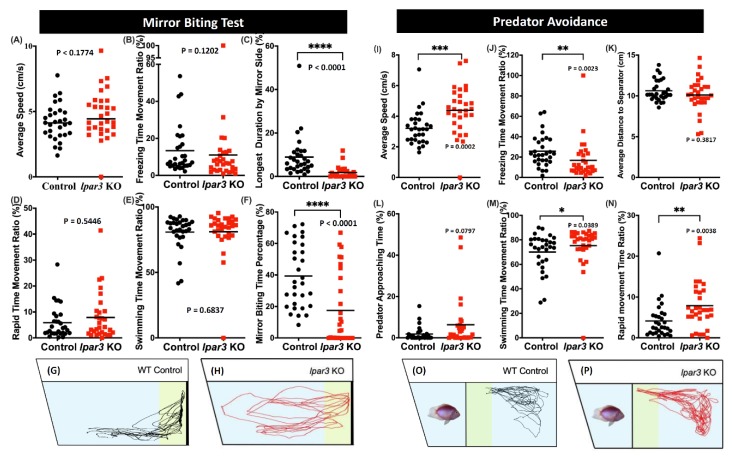
Comparison of behavior endpoints between wild-type (black) and *lpar3* KO (red) fish in mirror biting (**A**–**H**) and predator avoidance (**I**–**P**) tests. In the mirror-biting test, average speed (**A**), freezing time movement ratio (**B**), longest duration in the mirror side (**C**), rapid time movement ratio (**D**), swimming time movement ratio (**E**), and mirror biting time percentage (**F**) were analyzed. Locomotor trajectories of wild-type (**G**) and *lpar3* KO fish (**H**) for mirror biting assay were plotted to facilitate visualization. Average speed (**I**), freezing time movement ratio (**J**), average distance to the separator (**K**), predator approaching time (**L**), swimming time movement ratio (**M**), and rapid movement ratio (**N**) were analyzed during the predator avoidance test. Locomotor trajectories of wild-type (**O**) and *lpar3* KO fish for predator avoidance assay were plotted to facilitate visualization. The data are expressed as the median and were analyzed by Mann–Whitney test (*n* = 30 for both groups; * *p* < 0.05, ** *p* < 0.01, *** *p* < 0.001, **** *p* < 0.0001).

The mirror biting test was performed to evaluate the aggressive behavior of *lpar3* KO zebrafish [41]. The zebrafish aggression degree was measured by observing interactions with their image in the mirror. It was found that *lpar3* KO zebrafish exhibited significantly less aggression than the control group as supporting by the decreased mirror biting time percentage (Figure 3F) and the decline of the longest duration at mirror side observed in *lpar3* KO zebrafish (Figure 3C). However, mutant fish showed similar behavior with the control fish in terms of their locomotor activity during this test (Figure 3A,B,D,E). The locomotion trajectories are presented in Figure 3G,H; the yellow-colored region represents the area close to mirroring side, in which the control group had a higher approaching duration (Figure 3G) and *lpar3* KO larvae showed a more even path throughout the whole tank with no special interest in biting the mirror reflection (Figure 3H).

Next, the fear level was measured through the predator avoidance test. The zebrafish were incubated with a predator, convict cichlid (*Amatitlania nigrofasciata*), in the same tank divided by a transparent separator. Then, six fear-related endpoints were measured to observe the fish response to the presence of a predator. After the experiment, no alteration in the predator avoidance behavior was found in the mutant fish as there were no significant differences found in the predator approaching time and average distance to the separator of the mutant fish and the control fish (Figure 3L,K). Interestingly, hyperactivity-like behavior was observed in the mutant fish when they faced the predator, which is supported by the increased average speed, swimming time movement ratio, and rapid movement ratio, and decreased freezing time movement ratio (Figure 3I,J,M,N).

### 2.4. lpar3 KO Zebrafish Displayed Less Social Interaction and a Loose Shoaling Pattern

**Figure 4:**
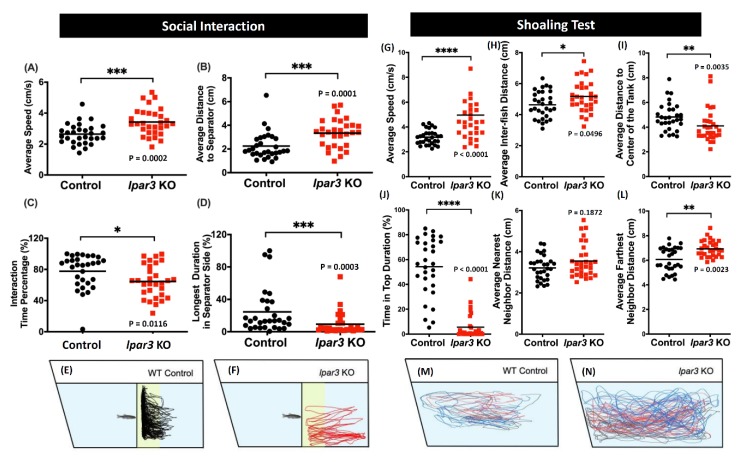
Comparison of behavior endpoints between wild-type (black) and *lpar3* KO (red) fish in social interaction (**A**–**F**) and shoaling (**G**–**N**) tests. In the social interaction test, average speed (**A**), average distance in separator side (**B**), interaction time percentage (**C**), and longest duration in separator side (**D**) were analyzed. Locomotor trajectories of wild-type (**E**) and *lpar3* KO fish (**F**) for social interaction assay were plotted to facilitate visualization. (**G**) Average speed, (**H**) average inter-fish distance, (**I**) average distance to the center of the tank, (**J**) time-in-top duration, (**K**) average nearest neighbor distance, and (**L**) average farthest neighbor distance were analyzed during the shoaling test. Locomotor trajectories of wild-type (**M**) and (**N**) *lpar3* KO fish for the shoaling test were plotted to facilitate visualization. The data are expressed as the median and were analyzed by nonparametric Mann–Whitney test (*n* = 30 for both groups; * *p* < 0.05, ** *p* < 0.01, *** *p* < 0.001, **** *p* < 0.0001).

Since zebrafish are highly social animals, the potential change in their social interaction caused by the *lpar3* gene knockout was interesting to observe [41]. In this experiment, we determined their social behavior through social interaction and shoaling tests. The social interaction of the zebrafish was assessed by observing their interaction with conspecifics [41]. Based on the results, *lpar3* KO fish showed significantly disrupted ability to interact with conspecifics, indicated by increased average distance to the separator and reduced interaction time percentage and longer duration in the separator side (Figure 4B–D). In addition, increased locomotor activity was also displayed by the mutant fish, supported by a higher average speed compared to the control group (Figure 4A). Summary of locomotor trajectories clearly revealed that *lpar3* KO fish showed less tendency to approach the separator than did those of the control (Figure 4F) while the wild type fish spent most of the time by the separator, indicated with the yellow color region (Figure 4E).

Furthermore, the social interaction of multiple zebrafish was evaluated through the shoaling test. It was observed that the mutant fish formed a loose shoal as indicated by a higher average inter-fish distance and an increased average farthest neighbor distance compared to the control group, even though there was no significant difference in their average nearest neighbor distance (Figure 4H,K,L). In addition, the locomotor trajectories revealed clearly that *lpar3* KO fish moved in a rather large area with three individuals further separated. This alteration was also implied by the abnormal locomotor activity and exploratory behavior of the mutant fish, which was shown by the increased average speed, the reduced average distance to the center of the tank, and reduced time-in-top duration (Figure 4G,I,J). From the trajectory tracking, *lpar3* mutants showed a larger swimming area across the whole tank (Figure 4N) compared to the control wild type (Figure 4M).

### 2.5. lpar3 KO Zebrafish Displayed Circadian Rhythm Locomotor Activity Dysregulation

**Figure 5:**
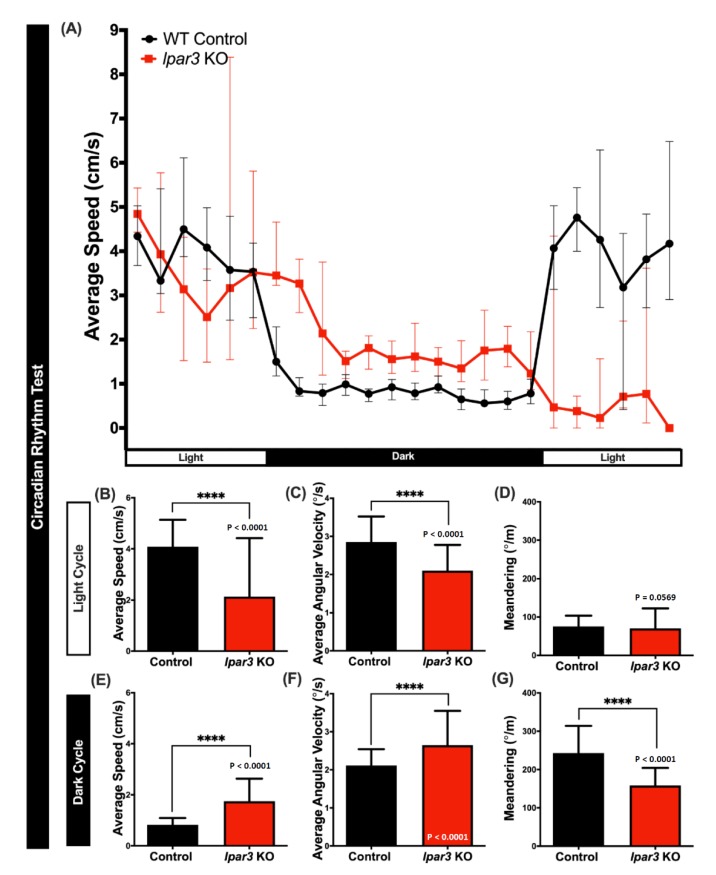
Comparison of behavior endpoints between wild-type (black) and *lpar3* KO (red) fish in the circadian rhythm locomotor activity assay. (**A**) Comparison of chronological changes of the average speed in light and dark cycles. Comparison of the average speed (**B**), average angular velocity (**C**), and meandering (**D**) between wild-types and *lpar3* KO fish in the light cycle. Comparison of the average speed (**E**), average angular velocity (**F**), and meandering (**G**) between wild-type and *lpar3* KO fish during the dark cycle. The data are expressed as the median with interquartile range and were analyzed by the nonparametric Mann–Whitney test (*n* = 18 for control; *n* = 16 for *lpar3* KO fish; **** *p* < 0.0001).

Since zebrafish are a typical diurnal species, they display robust locomotor activity during the day and sleep-like behavior during the night [42]. In this test, the association of the *lpar3* gene with the circadian rhythm locomotor activity of the zebrafish was tested by measuring three behavioral endpoints, namely the average speed, average angular velocity, and meandering for each light and dark cycle. From the results, it was observed that *lpar3* KO fish displayed dysregulated circadian rhythm locomotor activity. In the light cycle, less average speed and average angular velocity (Figure 5A,B) indicated irregular movement orientation and reduced locomotor activity, which was consistent with the result of the 3D locomotor activity and novel tank test. However, there was no significant difference in meandering observed during the light cycle (Figure 5D). Furthermore, an irregular pattern of mutant fish activity was also detected during the dark cycle, which was illustrated by higher average speed and average angular velocity, and reduced meandering observed in the *lpar3* KO fish (Figure 5E–G). Taken together, *lpar3* mutants had higher activity than the control fish in the dark cycle and lower activity in the light cycle, indicating rather extreme locomotion compared to the wild type.

### 2.6. lpar3 KO Zebrafish Displayed Reduction of Color Preference

**Figure 6:**
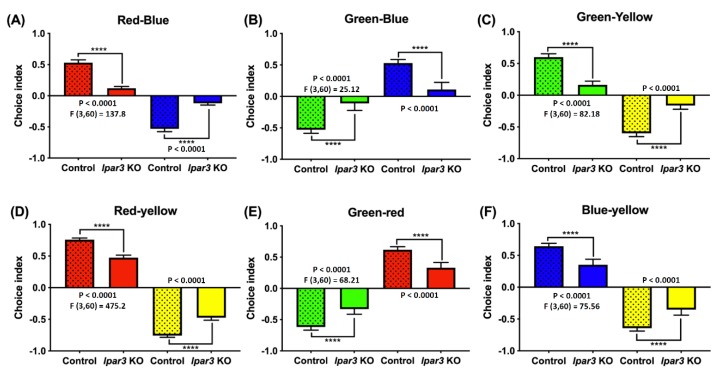
Comparison of color preferences between wild-type and *lpar3* KO zebrafish. The color preference results obtained from (**A**) red and blue combination, (**B**) green and blue combination, (**C**) green and yellow combination, (**D**) red and yellow combination, (**E**) green and red combination, and (**F**) blue and yellow combination. The data are presented with means ± standard error of the mean (SEM) and analyzed by one-way ANOVA, followed by Tukey multiple comparison analysis with (*n* = 24; ns = non-significant; * *p* < 0.05; ** *p* < 0.01; *** *p* < 0.001; **** *p* < 0.0001).

Previous research demonstrated that LPA is a potent compound that guides the retinal axons during development. The primary retinal neurons may be affected by neuronal inhibitory effects such as growth cone collapse and neurite retraction. The *lpar3* receptor plays a small but significant role in growth cone collapse [43]. Therefore, in this study, a color preference test was done to investigate whether *lpar3* gene deficiency would affect the color preference and perception of zebrafish. Unfortunately, there are still some differences regarding zebrafish color preference ranking based on several previous studies that may be caused by the differences in the color wavelength, light source position, and luminescence applied in the prior studies [44,45,46,47,48,49,50,51]. However, before conducting the experiment, considerable numbers of wild-type fish were tested to observe their color preferences ranking in our test with the current setting. Typically, based on our results, WT zebrafish in normal conditions have a clear color preference ranking as follows: red > blue > green > yellow and this color preference ranking sequence remained in the *lpar3* KO zebrafish. However, a significant reduction in color preference index was observed in the *lpar3* KO zebrafish compared to the wild-type (WT) fish. In red and blue (Figure 6A), red and green (Figure 6D), red and yellow (Figure 6E) combinations, the red preference index was significantly decreased compared to WT fish. *lpar3* KO fish also displayed a blue preference index reduction when combined with green (Figure 6B) and yellow (Figure 6F) in comparison with the WT fish. In the green and yellow combination test, the green preference index of *lpar3* KO fish was significantly reduced compared to that of the WT fish. The results show the same color preference in both the control and KO groups, but show a lower tendency of color approaches.

### 2.7. lpar3 KO Zebrafish Reduced Short-Term Memory Retention

**Figure 7:**
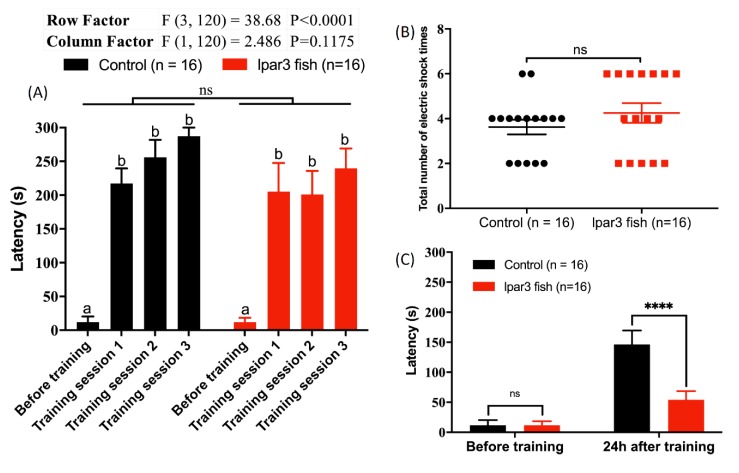
Comparison of the short-term memory between wild-type (WT) and *lpar3* knockout zebrafish by passive avoidance test. (**A**) Comparison of the learning latency between WT (black color) and *lpar3* KO fish (red color) moving into the black chamber with electric shock presence. (**B**) Comparison of the total electric shock number given for training between WT (black color) and *lpar3* KO fish (red color). (**C**) Comparison of the memory latency between WT (black color) and *lpar3* KO fish (red color) moving into the black chamber. Data are presented as means ± standard error of the mean (SEM) (*n* = 16) and the significant difference was tested by two-way ANOVA in (**A**) and (**C**), and by *t*-test in (**B**) (*n* = 16; ns = non-significant; **** *p* < 0.0001). Different letters (a, b) on the error bars represent significant differences (*p* < 0.05). The fish in this test were 8 months old.

A passive avoidance test in the shuttle box was performed to assess whether *lpar3* deficiency could impair short-term memory in adult zebrafish. *lpar3* KO zebrafish showed no significant difference in both learning latency (Figure 7A) (F_1120_ = 2.486, *p* = 0.1175) and the total number of electric shocks given for training (Figure 7B). However, most of the *lpar3* KO fish received a higher number of electric shocks in comparison to the WT fish, thereby indicating that the *lpar3* KO fish are more difficult to train than the WT fish. The latency after the training day of *lpar3* KO fish was significantly decreased compared to the WT fish, which showed no difference (Figure 7C). These results clearly indicate that *lpar3* deficiency could be related to short-term memory impairment in adult zebrafish.

### 2.8. ELISA Assay for Monitoring Biomarker Expression in the Brain and Whole Body

After the behavioral tests, the zebrafish were euthanized for the determination of stress hormone and neurotransmitter levels in the brain and muscles. ELISA kits were used to quantify the biomarkers related to stress, such as cortisol, and levels of neurotransmitters, such as serotonin (5-HT), melatonin, norepinephrine (NE), epinephrine (EPI), acetylcholine (ACh), acetylcholinesterase (AChE), and γ-aminobutyric acid (GABA). After extensively examining the hormone content in both brain or body tissues by ELISA with a high biological repeat number (*n* = 6 to 12), no significant difference of biomarker content was found in either brain or muscle tissues between control and *lpar3* KO fish (*t* test, *p* value = 0.096 to 0.981) (Table 1).

### 2.9. Hierarchical Clustering and PCA Analysis of Several Mutant Zebrafish Behavioral Endpoints

**Figure 8:**
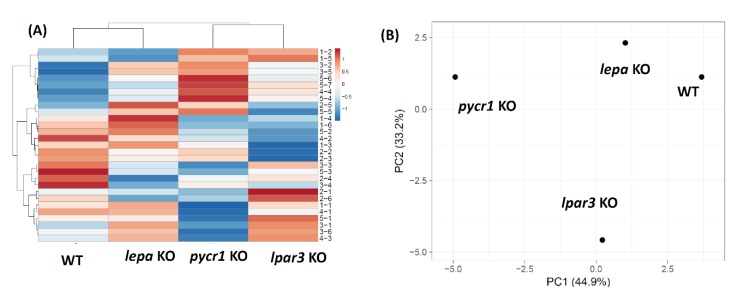
(**A**) Hierarchical clustering analysis and (**B**) principal component analysis (PCA) of multiple behavior endpoints in *lpar3* KO fish. In addition, other mutant zebrafish behavioral data from previous publications of *lepa* KO and *pycr1* KO fish were included to compare the behavior alteration patterns of each mutant fish group. WT, wild-type fish.

Next, to explore the behavioral phenomics between *lpar3* KO fish and other previously studied mutant fish, principal component analysis (PCA), hierarchical clustering, and heatmap comparison were performed based on five different zebrafish behavioral tests, which included the novel tank, mirror biting, predator avoidance, social interaction, and shoaling tests. From the result, two clusters were generated, revealing the degree of similarity between *lpar3* KO with *pycr1* KO fish and distinct levels of behavior alteration of *lpar3* KO fish compared to the wild-type and *lepa* KO fish (Figure 8A). This hierarchical clustering is plausible since *lpar3* KO fish exhibited similar altered behavior at that of *pycr1* KO fish that were studied previously [52]. These behavior-alteration similarities include loss of exploratory behavior, conspecifics social interest, and ability to form a normal shoal, all of which were observed in the *lepa* KO fish [53]. However, even though *lpar3* KO and *pycr1* KO fish belong in the same cluster, they still possessed different patterns regarding their behavior endpoints, which is marked by red and blue colors in the heat map, indicating a significant increase and decrease of endpoint behavior values. Within the first cluster, relatively normal endpoint values (white color) were reported, whereas in the *pycr1* KO, relatively high behavior endpoints were shown (red color). After further investigation, we found that most of these alterations in the first cluster lay in the shoaling test results. This result may be explained by the looser shoal formed by the *pycr1* KO fish compared to the *lpar3* KO fish, even though both of these mutant fish were unable to form a normal shoal. The pattern differences were also observed in the middle cluster, where relatively low endpoint values (blue color) were shown by the *lpar3* KO, which is quite different from the *pycr1* KO fish, which possessed normal endpoint values (white color). Later, it was found that these dissimilarities were mainly caused by the differences regarding their level of aggressiveness, which was shown to be greatly reduced in the *lpar3* KO fish. Last, a different pattern is also shown in the last cluster. In this cluster, low-level endpoint values were shown by the *pycr1* KO fish (blue color) while, on the other hand, *lpar3* KO fish possessed high-level endpoint values (red color). Afterward, we found that higher locomotor activity of *lpar3* KO fish than *pycr1* KO fish observed during the tests led to these differences. Supporting this result, PCA also shows a close distance between wild-type and *lepa* KO fish, leaving *lpar3* KO and *pycr1* KO fish on two distant sides, individually (Figure 8B). This gap between the *lpar3* KO and *pycr1* KO fish may be due to the different patterns of the behavior endpoints that are displayed in the heatmap. Taken together, even though there are several differences regarding their behavior, *lpar3* KO fish are more comparable to the *pycr1* KO fish in terms of behavior than to the *lepa* KO fish and control fish. Furthermore, for the first time, this result highlights the uniqueness of the behavioral pattern of zebrafish with an *lpar3* deficit. In the future, this finding may be fundamental to the study of other LPAR genes and their effects in terms of zebrafish behaviors. The legend of the behavior endpoints from the heatmap can be found in Table A1.

## 3. Discussion

As an extracellular lipid mediator, LPAR plays an important role on modulating multiple biological activities through the activation of G protein-coupled receptors. LPAR is known as a heteromeric G protein-coupled receptor (GPCR) specifically involved with G(i)/G(o)/G(q), and six LPA receptor subtypes (LPAR1/Edg2, LPAR2/Edg4, LPAR3/Edg7, LPAR4/GPR23/P2Y9, LPAR5/GPR92, and LPAR6/P2Y5) have been identified [54]. The expression pattern and signaling properties of LPARs are complex and result in multiple influences on physiological, pathological, and developmental processes, which in turn act through GPCR. Among multiple LPAR subtypes, our research group is more interested in LPAR3 since it as one of the members of the endothelial differentiation gene family regulating differentiation and development of the circulation system [55]. Our research group identified LPAR3 as playing a role in erythropoiesis induction [55] and megakaryopoiesis inhibition [53]. Those works concluded that LPAR3 induced an EPO-dependent erythropoietic process through the activation of LPAR3 and β-catenin. Therefore, LPAR3 may participate in the process to provide a novel treatment for promoting erythropoiesis [55]. In addition, LPAR3 has also been reported to play a role in embryo implantation and spacing in mice [56], mediate chemotaxis of immature murine dendritic cells to unsaturated LPA [57], regulate cellular functions during tumor progression in pancreatic cancer cells [58], and modulate the aging process [37].

**Figure 9:**
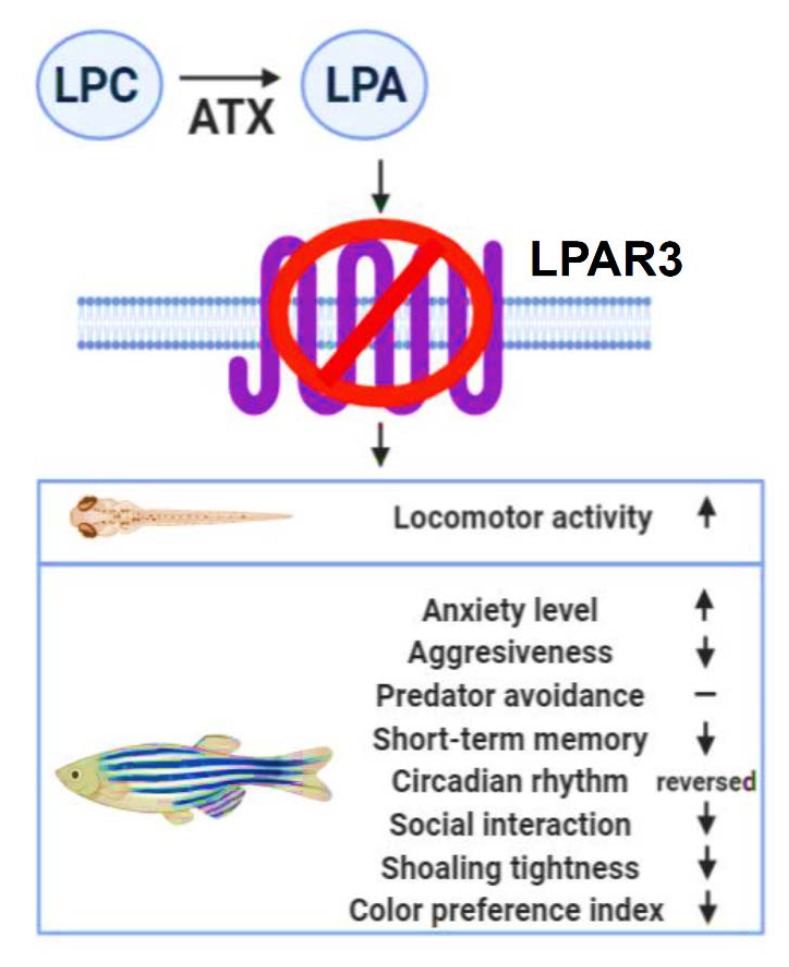
Schematic representation of the behavioral signatures detected in *lpar3* knockout zebrafish. LPA converted by autotaxin (ATX) from lysophosphatidylcholine (LPC), mediates numerous signaling pathways through LPA receptors. The novelty of this study was to identify that LPAR3, one of LPARs, plays multiple roles in modulating behaviors in zebrafish at both the larva and adult stages.

In addition to its function on embryogenesis, LPAR signaling was identified as playing a role in behavior control in a recent study. By a gene targeting approach, the LPAR1 has been reported as a requirement for normal suckling behavior in mice [36]. By an antagonist approach, LPA has been identified to induce anxiety-like behavior via its receptors in mice [59]. In addition, the LPAR5 has been reported to play a role in pain sensitivity, emotional exploration, and reversal learning [32]. All these previous findings suggest LPA signaling might also play a role in behavioral control in vertebrates. Based on previous behavior-related functions of LPAR reported in rodents, in this study, we aimed to investigate whether *lpar3* also plays an important role in behavioral control. By a locomotor test in larvae, we found *lpar3* KO larvae showing significant locomotor hyperactivity. This interesting finding encouraged us to further explore the potential role of *lpar3* on behavior control in adult fish by conducting a panel of behavioral tests including 3D locomotion, novel tank, mirror biting, predator avoidance, social interaction, shoaling, circadian rhythm locomotor activity, color preference behavior, as well as short-term memory tests. Based on the results, *lpar3* KO zebrafish exhibited abnormal exploratory behavior with a relatively high anxiety level, which was observed in the novel tank diving assay. In agreement with these results, this anxiety-like phenotype was also observed in LPAR1-deficient mice [33]. The LPAR1 mutant mice exhibited a reduced exploratory behavior when exposed to a novel environment in the open field exploration and elevated plus maze tests. The impaired exploration could be a consequence of the concomitant anxiety levels during the test. Surprisingly, the observed anxiety-like phenotype found in LPAR1-deficient mice and the current results were opposite to the observed anxiolytic phenotype in LPAR5-deficient mice. Signs of reduced anxiety were revealed in the LPAR5-deficient mice after several exploratory tasks [32,33]. In addition, while there was no significant alteration on predator avoidance behavior, a significantly compromised aggression level was observed in *lpar3* KO zebrafish in comparison to the control group. Moreover, a significant decrease in the ability to interact with conspecifics by showing a low level of interaction time percentage was also identified in *lpar3* KO fish. In addition, there was also a reduction in shoaling tightness in the shoaling test. Interestingly, alterations in social-related behaviors were also displayed by LPAR5-deficient mice, after marked reduction in social exploration was observed when they encountered novel conspecifics [32]. Furthermore, *lpar3* KO zebrafish also displayed severe dysregulation in circadian rhythm locomotor activity showing relatively high locomotion in the night cycle and lower activity during the day cycle. This finding was found to be similar with a previous study done by Vegh and colleagues. In their study, LPAR5-deficient mice showed nocturnal hyperactivity in the cage activity test [32]. Next, a significant reduction in short-term memory retention in *lpar3* KO zebrafish was also identified (behavioral alterations are summarized in Figure 9). This result is similar with the previous study in LPAR1-deficient mice. LPA1-deficient mice exhibited a clear deficit of reference memory during the last day of training when these mice were unable to achieve the performance level attained by the wild-type mice. Moreover, deficit of spatial working memory was also reported in these mutant mice [33]. Taken together, our behavioral outcomes support previous studies that suggest a hippocampal malfunction in the absence of the LPAR3, even though LPAR3 is not the primary LPAR expressed in hippocampal neural progenitor cells in mice [60]. Furthermore, behavior alterations observed in the mutant fish may also relate to neurite branching since another prior study in mice neurons found that this important process for neuronal network formation was induced through the introduction of LPAR3 and the addition of LPA into hippocampal cell cultures [61]. Additionally, the behavioral results in the current study may also be supported by a previous report in rodents showing temporal expression changes for three LPA receptor subtypes (LPAR1–LPAR3) in two different injury models: traumatic brain and spinal cord injuries [62]. In summary, the findings of this study provide strong evidence to support for the first time that *lpar3* plays an important role in modulating multiple behaviors in vertebrates.

The temporal and spatial distributions of neuromediator systems have been characterized in both larval and adult zebrafish for some of the key mediators implicated in psychiatric diseases such as GABA, glutamate, ACh, dopamine, and serotonin [63]. The neurochemistry of vertebrates is highly conserved [64,65,66] and alteration in neurotransmission is one of the main causes of psychiatric disorders [67,68]. Zebrafish have been known to possess all primary neuromediator systems including transporters, receptors, transmitters, and enzymes required for the synthesis and metabolism of the mediators [69]. By ELISA, we measured the relative content of neurotransmitters and hormones by total tissue weight and extraction volume in brain and muscle tissues, but no significant difference was identified for those biomarkers at protein/molecular levels. The consistent levels of stress hormone and neurotransmitters identified in *lpar3* KO fish brain and body tissues is surprising. The following possibilities are proposed to explain this observation: (1) The *lpar3* expressing level in the brain might be very low and restrictedly distributed. (2) The relative amount of neurotransmitter or hormone affected in *lpar3*-deficient zebrafish brain or body tissues may be diluted and cannot be distinguished by ELISA. Further studies of *lpar3* in situ through hybridization in brain sections or performing ELISA tests in different brain regions are necessary to test these possibilities. However, in our mix-gendered study, there were limitations in the lack of a significant difference in the ELISA results. As previous studies illustrated gender differences in the cerebellar metabolism of humans [70] and male-female differences in swimming pattern behavior of zebrafish [71], sex-dependent characteristics in neural and locomotion have been suggested. The possibility of gender-specific research in the future might lead to a novel finding from our study.

There were several important neurotransmitters that consistently showed a reduction trend in both brain and body tissues; these were norepinephrine (NE) and epinephrine (although the trends did not reach significant levels). NE is a hormone controlling mobilization between brain and muscle and this hormone has been shown to be the major adrenergic mediator at postganglionic neuroeffector junctions [72]. Previous studies have shown that a low level of NE was found to cause depression and lack of motivation in animals [73,74]. In line with this prior study, zebrafish behavior alterations were observed in the mutant fish, such as reduced aggression and social interaction, and these phenomena may be related to the slightly lower levels of NE possessed by the *lpar3* KO fish. Meanwhile, as a predominant sympathomimetic amine released by the adrenal medulla, epinephrine is also associated with aggressive responses. In addition, epinephrine has been related to a wide variety of emotional responses to stressful situations [72]. If a fish is exposed to a stressor, it may have initial behavioral changes, followed by primary, secondary, and tertiary stress responses. Changes in epinephrine and norepinephrine may begin within the primary response [75]. A previous study in murrel showed disruption of brain epinephrine and norepinephrine levels after exposure to carbofuran was localized to the region of the brain regulating behavior and motor activity, suggesting preferential alteration of swimming behaviors [76]. Moreover, neuronal function of LPAR3 has been demonstrated in a previous study; LPA introduced neuronal cells show higher neurite branching through LPAR3 involvement [25]. In the present study, this alteration was shown by the hyperactivity-like behavior exhibited by the mutant fish. Besides these two neurotransmitters, serotonin, which also was found to be slightly upregulated in both tissues, may also play a role in the *lpar3* KO fish behavior alteration, since serotonin, in particular, has been implicated in both epinephrine and cortisol regulation in fish during stress [77].

Due to easy handling and control measures, zebrafish are an efficient model organism for pharmacological studies, including for long-term memory disturbances using pharmacological drugs that inhibit protein synthesis. Moreover, since it is easy to deliver the substance of interest to the zebrafish, mutant fish can demonstrate more alteration patterns based on zebrafish cognition studies, as the ACh system in zebrafish aging reflects human aging [78]. ACh is known to play a key role in the central nervous system. It is involved in sustaining attention, memory/learning processes, and sleep. Therefore, ACh dysregulation may lead to major distress and diseases such as attention deficit hyperactivity disorder (ADHD), anxiety, and schizophrenia. In this study, although the change did not reach a significant level, the ACh levels were lower in the brain of the *lpar3* KO zebrafish group as compared to that of the control group, which can be associated with anxiety-like behavior, as shown by the abnormal exploratory behavior and hyperactivity-like behavior displayed in the behavior tests, and the loss of memory observed in the mutant fish. In line with the present results, the previous study found a memory impairment in zebrafish after being incubated with 111 mM glucose for 2 weeks, accompanied by ACh dysregulation [79]. The passive avoidance test for *lpar3* KO fish supports *lpar3* function as it is associated with memory retention in zebrafish. In addition, hyperactivity-like behavior observed in the *lpar3* KO zebrafish during the dark cycle may be related to melatonin, a hormone that plays a key role in regulating circadian rhythms [80,81,82]. As discussed before, the levels of this related neurotransmitter must be carefully determined in the subregions of brains to reveal further evidence. The results of the behavioral tests indicated that the *lpar3* KO zebrafish displayed anxiety-related behavioral abnormalities, therefore, another possibility is that *lpar3* functions on particular anxiety-related neurocircuits. In conclusion, based on extensive behavioral tests, our results show for the first time that the *lpar3* gene plays a role in modulating behaviors of anxiety, aggression, social interaction, circadian rhythm locomotor activity, and memory retention in zebrafish. This interesting finding opens a new avenue for the future study of how LPAR3 or other LPARs affect behavior in rodents or humans.

## 4. Materials and Methods

### 4.1. Animal Ethics

All experimental protocols and procedures involving zebrafish were approved by the Committee for Animal Experimentation of the Chung Yuan Christian University (Number: CYCU104024, issue date 21 December 2015). All experiments were performed in accordance with the guidelines for laboratory animals. Wild-type AB strain and *lpar3* KO zebrafish were maintained in a recirculating aquatic system at 28.5 °C. Circulating water in the aquarium was filtered by reverse osmosis (pH 7.0–7.5). The zebrafish were fed twice a day with lab-grown brine shrimp. Six- to eight-month-old zebrafish of mixed gender were used for the behavioral tests.

### 4.2. Generation of lpar3 knockout fish by TALEN

The commercial TALEN plasmid was purchased from Zgnebio Co, Taipei, Taiwan. *Escherichia coli* Dh5α was used to amplify the plasmid-containing left and right arm RVD modules and the purified plasmids were linearized with NotI. Following the in vitro transcription by mMESSAGE mMACHINE SP6 transcription kit (Invitrogen Ambion, Waltham, MA, USA) at 37 °C for two hours, LiCl precipitation was performed to retrieve the TALEN mRNAs. Then, the TALEN mRNA for left and right arms were mixed for microinjection into the embryos. Different gradients of TALEN mRNA mixture were injected into 1–2 cell embryos, and embryos were collected for DNA purification. After PCR amplification at the targeting site by gene-specific primers, the potential insertion/deletion was verified by the T7E1 endonucleases digestion. The uncut PCR band referred to successful insertion/deletion and the corresponding sequences were validated Sanger sequencing.

### 4.3. Zebrafish Larvae Light/Dark Cycle Test

Forty-eight zebrafish larvae at ~5 dpf were transferred to 48-well plates individually and placed in a high throughput monitoring Zebralab (ViewPoint Life Sciences, Lyon, France) chamber for light and dark cycles test recording. After ~30 min of an acclimation phase, the light and dark cycles with 10 min intervals of each cycle were started and were repeated four times. A total of 80 min of recording with swimming distance data were collected. Swim speed thresholds were set based on a previous study to define three different speed thresholds. These speeds included bursting (>2.0 cm/s), which were short, intermittent, and powerful bouts of activity, cruising (0.5 > s < 2.0 cm/s), covering most of the commonly measured larval speeds, and freezing (<0.5 cm/s) during which larvae displayed minimal activity [83]. Later, the video was analyzed further to obtain the total burst and rotation counts by Zebralab software.

### 4.4. Zebrafish Adult Behavioral Assessment

The behavioral analyses, excluding the circadian rhythm locomotor activity test, were performed in the morning until the early afternoon (9:00 to 15:00) and started with a five- to ten-minute pre-acclimation in the test tank, except for the novel tank test. The behavior tests were as follows: novel tank, shoaling, mirror biting, social interaction, predator avoidance, color preference, and three-dimensional (3D) locomotion tests. Three-dimensional locomotor activity was measured by using an acrylic tank (20 cm × 20 cm × 20 cm) with ~6 L water according to a previously described protocol [39]. The novel tank, mirror biting, predator avoidance, social interaction, and shoaling tests were performed by a designed zebrafish tower consisting of ten trapezoid test tanks (28 cm × 5 cm × 15 cm) with ~1.25 L water in each according to our previously published protocols [40]. For the circadian rhythm locomotion activity test, a designed chamber equipped with an infrared camera was used following the method described in a previous publication [42]. The one-minute videos were recorded every hour for 24 h (12:12 h light/dark). Later, the recorded videos were analyzed by idTracker to extract the X and Y axes locomotor coordination [84].

### 4.5. Color Preference Test

Acrylic tanks with a size of 21 cm × 21 cm × 10 cm were divided into two sections to compare a set of two color combinations at once. Four different colors, red, green, blue, and yellow, were assembled two-by-two for a total of six combinations. The recording was conducted with a high-resolution camera following with analysis by idTracker. Fish activity was recorded for 30 min while two different background colors were placed underneath the transparent tank; the relative time spent over each color was calculated for comparison.

### 4.6. Passive Avoidance Test (Short-Term Memory Test)

The device for the passive avoidance test was constructed by a 30 cm × 20 cm × 20 cm acrylic tank with two compartments, black and white, of equal size separated by a gate. At first, a fish was placed in the tank with the gate open for 5 min for environmental recognition. Then, the fish was moved to the white area during a training session for one minute with the gate closed for acclimation. Later, the gate was opened again allowing the fish to enter a dark area. After the fish crossed the gate to the dark side, a mild electric shock (25 V, 1 mA) was administered up to three times with a 5-s interval. The fish was moved back to the white side if it did not swim back with 5 min of observation. The training was repeated a maximum of three times with one-minute acclimation in between. After 24 h, the fish was placed in the white area to test the time it took to enter the dark compartment, as a short-term memory indicator.

### 4.7. Determination of Biomarker Expression by ELISA

After the behavioral tests, both male and female zebrafish were euthanized using iced water. Then, the brain, and body tissues were isolated and subjected to protein extraction. All of the tissue samples were placed on ice during collection. With the addition of ten (10) volumes (*v*/*w*) of ice-cold phosphate saline buffer, samples were homogenized with a Bullet blender (Next Advance, Inc., Troy, NY, USA) at 4 °C. After 30 min on ice, the samples were centrifuged at 13,000 rpm for 20 min at 4 °C. The supernatant was collected and stored at −80 °C for further experiments. Protein samples were compared with the standard of Pierce BCA (bicinchoninic acid) Protein Assay Kit to determine the total protein concentration (23225, Thermo Fisher Scientific, Waltham, MA, USA). The protein absorbance was read at 562 nm with a microplate reader (Multiskan GO, Thermo Fisher Scientific, Waltham, MA, USA). Different neurotransmitters and hormones, such as epinephrine (ZGB-E1589), norepinephrine (ZGB-E1571), melatonin (ZGB-E1597), cortisol (ZGB-E1575), acetylcholine (ZGB-E1585), acetylcholinesterase (ZGB-E1637), serotonin (ZGB-E1572), and GABA (ZGB-E1574) were measured by commercial target-specific kits (Zgenebio Inc., Taipei, Taiwan). Tissue samples were diluted in 96-well microplates coated with specific antibodies, followed by the addition of conjugate solution and incubation for one hour at 37 °C. After the removal of unspecific bindings with washing buffer, the homogenates and HRP-conjugated antibodies were added to the 96-well microplates and incubated for 15 min at 37 °C. Then, the stop solution was added and the microplate was read at 450 nm with a microplate reader (Multiskan GO, Thermo Fisher Scientific, Waltham, MA, USA). The absorbance readings were calculated and the standard curve was generated from the standard readings.

### 4.8. PCA, heatmap, and clustering analysis

All of the behavioral endpoint values from five different tests (novel tank, mirror biting, predator avoidance, social interaction, and shoaling tests) were calculated as a comma separated values file (.csv) using Microsoft Excel. All of the important behavioral endpoints are listed in Table A1. Later, data were imported into ClustVis (https://biit.cs.ut.ee/clustvis/), a web tool designed for visualizing and clustering multivariate data. Since the data range covered multiple magnitudes, data transformation was performed during the preprocessing step. Afterward, data transformation by ln(x + 1) was conducted, because the smallest value on the data was 0 and to treat each variable equally, unit variance scaling for each row was carried out. Subsequently, SVD with the imputation method was used to calculate principal components as there were no missing values in the dataset [85]. Heatmap and PCA were exported and saved in the computer system after it was processed.

### 4.9. Statistical Analysis

All statistical analyses were plotted and calculated by using GraphPad Prism (GraphPad Software version 8 Inc., La Jolla, CA, USA). Each fish group was compared to the control group, using either *t*-test, Mann–Whitney, one-way, two-way ANOVA, or Kruskal–Wallis tests, depending on the data structure and normality for significance determination. A follow-up test, which was Sidak’s or Tukey multiple comparison test, was done. The significant difference between control and treated groups is marked as * if *p* < 0.05, ** if *p* < 0.01, *** if *p* < 0.001, and **** if *p* < 0.0001.

## Figures and Tables

**Table 1:** Expression of stress hormones and neurotransmitters in the brain and other body parts in wild-type and *lpar*3 knockout zebrafish at the adult stage. Data are presented as means ± standard error of the mean (SEM) and the significant difference was tested by t-test.

Biomarker	WT	*lpar3* KO	Unit	Significance	*p* Value
**Brain (*n* = 6 for all except GABA, *n* = 3 for GABA)**					
Norepinephrine	5.68 ± 1.174	4.00 ± 0.6009	pg/ug total protein	NO	0.221
Epinephrine	8.38 ± 1.892	6.28 ± 1.085	pg/ug total protein	NO	0.225
Melatonin	0.05 ± 0.006	0.03 ± 0.004	pg/ug total protein	NO	0.343
Cortisol	0.85 ± 0.115	0.70 ± 0.094	pg/ug total protein	NO	0.659
Acetylcholine	13.60 ± 1.381	12.18 ± 1.64	pg/ug total protein	NO	0.715
Acetylcholinesterase	5821.75 ± 1129	5026.09 ± 803.6	pg/ug total protein	NO	0.473
5-HT	82.00 ± 9.476	96.79 ± 12.02	pg/ug total protein	NO	0.614
GABA	444.042 ± 133.35	394.43 ± 700.03	ng/ug total protein	NO	0.599
**Body (*n* = 12 for all except GABA, *n* = 6 for GABA)**					
Norepinephrine	7.45 ± 1.379	7.04 ± 0.7961	pg/ug total protein	NO	0.096
Epinephrine	12.95 ± 2.011	12.61 ± 1.475	pg/ug total protein	NO	0.318
Melatonin	0.06 ± 0.009677	0.08 ± 0.009607	pg/ug total protein	NO	0.981
Cortisol	1.22 ± 0.204	1.43 ± 0.1851	pg/ug total protein	NO	0.752
Acetylcholine	22.84 ± 3.004	23.25 ± 2.3	pg/ug total protein	NO	0.389
Acetylcholinesterase	8791.82 ± 1086	9004.17 ± 1300	pg/ug total protein	NO	0.560
5-HT	143.29 ± 17.95	177.07 ± 23.01	pg/ug total protein	NO	0.422
GABA	712.55 ± 292.24	844.65 ± 311.36	ng/ug total protein	NO	0.466

## Appendix A

**Table A1:** Summary of zebrafish behavior endpoints measured from the novel tank, mirror biting, predator avoidance, social interaction, and shoaling tests.

Index	Behavior Endpoints (units)	Definition	Data Interpretation
1-1 2-1 3-1 4-3 5-1	Average speed (cm·s^−1^)	Total distance traveled divided by total time duration	Reflects general motor/neurological phenotypes
1-2 2-4 3-4	Freezing time percentage (%)	Total percentage of time when zebrafish speed was less than 1 cm·s^−1^	Indicates increased anxiety and freezing time is generally higher in stressed zebrafish
1-3 5-2	Time spent in top percentage (%)	Total time spent in the top portion of the tank	Increasing value indicates lower anxiety levels
1-4	Number of entries to the top	Total times zebrafish swims to the upper half of the tank	
1-5	Latency to enter top (s)	The amount of time it takes the fish to cross into the upper half of the tank	Increasing value indicates higher anxiety levels
1-6	Distance traveled in the top (cm)	Total distance traveled in the top portion of the novel tank	Increasing value indicates lower anxiety levels
2-2	Mirror biting time percentage (%)	Total percentage of time when zebrafish bit the mirror	Increasing value indicates higher aggression levels
2-3	Longest duration in mirror side percentage (%)	Total percentage of zebrafish longest duration stayed in front of mirror	
2-5 3-5	Swimming time percentage (%)	Total percentage of time when zebrafish speed was less than 1 cm·s^−1^	Increasing/decreasing value indicates higher anxiety levels
2-6 3-6	Rapid time movement percentage (%)	Total percentage of time when zebrafish speed was more than 10 cm·s^−1^	
3-2	Predator approaching time percentage (%)	Total percentage of time when zebrafish approached the predator	Increasing value indicates altered predator avoidance behavior
3-3	Average distance to predator separator (cm)	Average distance of zebrafish to the separator of the predator	Decreasing value indicates altered predator avoidance behavior
4-1	Zebrafish interaction time percentage (%)	Total percentage of time when zebrafish interacted with another zebrafish	Increasing value indicates higher sociability levels
4-2	Longest duration in separator side percentage (%)	Total percentage of zebrafish longest duration stayed in front of separator	
4-4	Average distance to predator separator (cm)	Average distance of zebrafish to the separator of the conspecific	Decreasing value indicates higher sociability levels
5-3	Thigmotaxis (cm)	The average distance of the zebrafish from the center of the tank	Decreasing value indicates lower anxiety levels
5-4	Average inter-fish distance (cm)	Average distance between the body center of every member of the shoal	Increasing/decreasing value indicates higher anxiety levels
5-5	Average shoal area (cm^2^)	Average size of the shoal	
5-6	Average nearest neighbor distance (cm)	Distance for the body center of each fish to the closest neighboring fish	
5-7	Average farthest neighbor distance (cm)	Distance for the body center of each fish to the farthest neighboring fish	
